# Oral health associated with incident diabetes but not other chronic diseases: A register-based cohort study

**DOI:** 10.3389/froh.2022.956072

**Published:** 2022-08-18

**Authors:** Pia Heikkilä, Leo Niskanen, Anna But, Timo Sorsa, Jari Haukka

**Affiliations:** ^1^Department of Oral and Maxillofacial Diseases, Faculty of Medicine, University of Helsinki and Helsinki University Hospital, Helsinki, Finland; ^2^Internal Medicine, Päijät-Häme Central Hospital Hospital and Universities of Helsinki and Eastern Finland, Lahti, Finland; ^3^Department of Public Health, Clinicum, University of Helsinki, Helsinki, Finland; ^4^Division of Periodontology, Department of Dental Medicine, Karolinska Institutet, Huddinge, Sweden; ^5^Faculty of Medicine and Health Technology, Tampere University, Tampere, Finland

**Keywords:** periodontitis, diabetes, oral infections, chronic diseases, chronic systemic diseases

## Abstract

**Introduction:**

Oral infectious diseases are common chronic oral diseases characterized by a chronic inflammatory condition. We investigated chronic oral diseases as potential risk factors for systemic chronic diseases, diabetes mellitus, connective tissue diseases, seropositive rheumatoid arthritis, ulcerative colitis, and Crohn's disease, as well as severe psychotic and other severe mental disorders.

**Methods:**

The cohort comprised 68,273 patients aged ≥ 29 years with at least one dental visit to the Helsinki City Health Services between 2001 and 2002. The cohort was linked to the data on death (Statistics Finland), cancer (Finnish Cancer Registry), and drug reimbursement (Finnish Social Insurance Institution) and followed until death or the end of 2013. The outcomes of interest were the incidences of chronic diseases measured starting with special refund medication, which means Social Insurance Institution partly or fully reimburses medication costs. Outcomes of interest were diabetes mellitus, connective tissue diseases, seropositive rheumatoid arthritis, ulcerative colitis and Crohn's disease, and severe mental disorders.

**Results:**

The mean follow-up time was 9.8 years. About 25% of the study population had periodontitis, 17% caries, over 70% apical periodontitis, and 9% <24 teeth at the start of follow-up. Diabetes was the only chronic systemic condition associated with oral health variables. Having 24 to 27 teeth was associated with a higher incidence rate ratio (IRR) (1.21, 95% confidence interval 1.09–1.33) compared to having 28 or more teeth; the IRR for having 23 or less was 1.40 (1.22–1.60). Having periodontitis (1.10, 1.01–1.20), caries (1.12, 1.01–1.23), or apical periodontitis (1.16, 1.04–1.30) is also associated with a higher risk of diabetes.

**Conclusion:**

Our epidemiological 10 years follow-up study suggests that the association exists between chronic oral diseases and diabetes, warranting close collaboration among patient's healthcare professionals.

## Introduction

The two major oral infectious diseases, dental caries and periodontitis, are common chronic oral infectious diseases characterized by a chronic inflammatory condition, and their progressions are influenced by multiple factors [1]. Severe periodontitis affects 10% to 15% of the global adult population [2]. Periodontitis is associated with an increased risk for several chronic systemic diseases such as diabetes [2], inflammatory bowel disease [3], cancer [4], and cardiovascular diseases through systemic often low-grade inflammation as the etiopathogenic link [5–7]. Caries can lead to the formation of apical periodontitis (AP), also capable of promoting and affecting the course of various systemic diseases. The prevalence of AP in Europe has been reported to affect 61% of individuals and 14% of teeth and increase with age [8]. There may be a moderate risk and correlation between some systemic chronic diseases and endodontic pathologies. AP has also been related to cardiovascular diseases and diabetes [9].

Low-grade systemic and tissue inflammations precede diabetes onset and are often linked to insulin resistance and the development of diabetes and its complications [10, 11]. Since effective therapy and management of the periodontal disease are well-established, it is important to know for future prevention and control of diabetes whether periodontitis indeed plays a role in the development of diabetes and its potentially fatal complications [12]. However, evidence from clinical trials and observational studies is still scarce, and more follow-up studies are required [13]. A review of the effect of periodontal disease on diabetes with four studies, in total 22,230 individuals, reported significant adverse effects of periodontal disease on glycaemic control, diabetes complications, and development of type 2 (and possibly gestational) diabetes [2]. Because the evidence was scarce and partly not generalizable, we called for large-scale studies with long follow-ups.

In a recent review of inflammatory bowel disease (IBD) and oral health, we found a higher risk of periodontal disease and worse oral health in IBD patients than in non-IBD ones. This meta-analysis included only case-control studies. We stated that longitudinal studies are needed to establish a link between IBD and periodontal disease [3].

Evidence from systematic reviews supports the association between PD and a higher risk of rheumatoid arthritis [14–16]. However, most epidemiologic outcomes derive from case-control studies with relatively small sample sizes. Additionally, some evidence from animal models suggests the connection between PD and rheumatoid arthritis [17, 18].

We hypothesized that oral health abnormalities could precede and/or promote tissue inflammation related to chronic systemic conditions. We, therefore, investigated the associations between oral health and the incidence of the following systemic conditions: diabetes, IBD, connective tissue disease, and psychosis. The rationale for choosing these diseases were that they are relatively common in the general population, and thus in the registered studies, the diagnosis is reliable and readily assessed. These disorders are also distinct clinical entities, but as a matter of fact, all these disorders may have some common origins, for example, tissue inflammation seems to characterize these all, even mental disorders. Further, patients with mental disorders are usually also socially disadvantaged and have poorer somatic health in general, therefore the inclusion of this diagnostic category serves also as an internal control of the findings making the results more robust in supporting the close relationship between glucose metabolism and periodontitis.

The setting is a population-based follow-up observational register study where the initiation of drug medication measures incidence for specific studied chronic conditions with documented reimbursement.

## Methods

### Study population

We used the data from the patient register of the Public Dental Service of the City of Helsinki to identify all individuals aged 29 years or more with at least one primary dental healthcare visit between 1 January 2001 and 31 December 2002. For these patients, follow-up data on deaths and causes of death were obtained from the register of deaths of Statistics Finland [19] through a computerized register linkage using the unique personal identification codes assigned to every resident in Finland. Along with the date of death, mortality data also included the cause of death coded according to the 10th revision of the International Classification of Diseases (ICD-10). In addition, data on socioeconomic status and education were obtained from Statistics Finland. The dental care data were also linked to the Drug Reimbursement Register of the Finnish Social Insurance Institution (SII). These drug prescription records, except for institutionalized patients, cover the entire study population. In Finland, patients with chronic or severe diseases, such as diabetes, are granted special reimbursement rights for outpatient medical treatment based on a physician's statement on their condition and need for medication [20]. The cancer diagnosis data, date of diagnosis, and ICD-O-3 code [21] were obtained from the Finnish Cancer Registry (FCR). The FCR database contains data on virtually all cancers diagnosed in Finland since 1953. The coverage and accuracy of the Finnish Cancer Registry data are excellent [22, 23].

Altogether 71,200 patients visited the Public Dental Service of Helsinki from 2001 to 2002. We restricted the study population to those who had no history of cancer at the first visit, who were alive 2 years after the first visit, and were with data on the number of teeth and other information on dental status. The dentist has done full mouth examinations, including cariological status and periodontal status (probing, bleeding on probing) and panoramic radiographs/intraoral radiographs. Malignant diseases can promote and modify the development and courses of systemic inflammatory diseases [24–27]. The size of the final study population was 48,609 individuals. The follow-up started 2 years after the first visit and continued until the occurrence of the outcome, 31 December 2013, or death, whichever occurred first.

### Outcomes

The outcomes of interest were the incidence of several chronic diseases measured by starting a special refund right for medication. Special reimbursements of drug expenses are given to patients who have a statement from their doctor attesting to their condition and need for medication [20]. We used both SII refund groups and specific ICD10 codes included in SII groups (Supplementary Table 1) as outcomes. The following diseases were included: diabetes mellitus (SII code 103), connective tissue diseases (202), seropositive rheumatoid arthritis (M05), ulcerative colitis and Crohn's disease (208), Crohn's disease (K50), ulcerative colitis (K51), and severe psychotic and other severe mental disorders (112). Severe mental disorders were included as negative outcome control [28]. Any individuals with prevalent refund rights were excluded when incidence was studied.

### The measure of exposure and potential confounders

We utilized data from dental visits in the follow-up period starting 2 years after the first visit. Dentists use the classification of the Finnish SSI to record treatment measures provided, and these codes were used here. These data include procedure codes of dental treatment (gingivitis, periodontitis, caries, endodontic, surgery, and prosthesis), and information on dental status presented by a number of teeth and oral health indices, such as primary caries (I), number of decayed teeth (DT), decayed/missing/filled teeth (DMFT), and need for periodontal treatment due to periodontal pockets (CPI = the Community Periodontal Index). Exposure to periodontitis was defined as a binary variable (no/yes) based on periodontitis treatment procedure codes [24, 25].

Among potential confounders in this study were socio-demographic characteristics, such as age, sex, statins at baseline, and socioeconomic status, which were available for the entire study population. Statistics Finland's professions were categorized into eight broader categories, including unknown, to represent socioeconomic status (SES). Statin (ATC code C10A) use in baseline was determined from prescription data of SII. To account for dental status other than periodontitis, we used number of teeth (0–23, 24–27, 28–32), indices I (0, 1–2, 3–4, ≥5), DT (0, 1–2, 3–4, ≥5), DMFT (0–13, 14–18, 19–23, ≥24 according to quartiles), and CPI (0–1, 2, 3–4), number of healthy sextants (0, 1, 2–4, 5–6), number of toothless sextants (0, 1–6), and indicators of different dental treatments (yes/no) [29, 30]. I, DT, DMFT, and CPI indices were defined by taking the maximum value of those recorded during the dental visits within 2 years after the first visit. The number of healthy sextants was specified according to the first visit, and the number of toothless sextants was selected by the minimum value. For part of the study population, however, health indices were not available because it is not routinely recorded at every visit; these appointments were defined as follow-up visits. We excluded these individuals from the study population.

### Statistical method

Incidence was described with incidence rates and modeled with the Poisson regression model, and results were reported as incidence rate ratios (IRR). The following explanatory variables were included: sex, age, socioeconomic status, usage of statins in baseline (no, yes), number of teeth, I index, D index, DMF index, CPI, periodontitis (no, yes), caries (no, yes), and endodontic caries (no, yes). All calculations were carried out using the R language [31].

### Patient involvement and ethical considerations

No patients were involved in setting the research question or the outcome measures, nor were they involved in the study's recruitment, design, or implementation. Patients were not asked to interpret or disseminate results. The Ethical Committee of the Faculty of Medicine, University of Helsinki, Finland (01/2014), reviewed the protocol. Data permits were received from the Social Insurance Institute (SII) (68/522/2014), the National Institute for Health and Welfare (THL/1295/5.05.05/2014), and Statistics Finland (TK-53-1290-14). According to Finnish law, this is a register-based study with anonymous data and no patient contact; thus no consent from anonymized patients were required.

## Results

**Figure 1:**
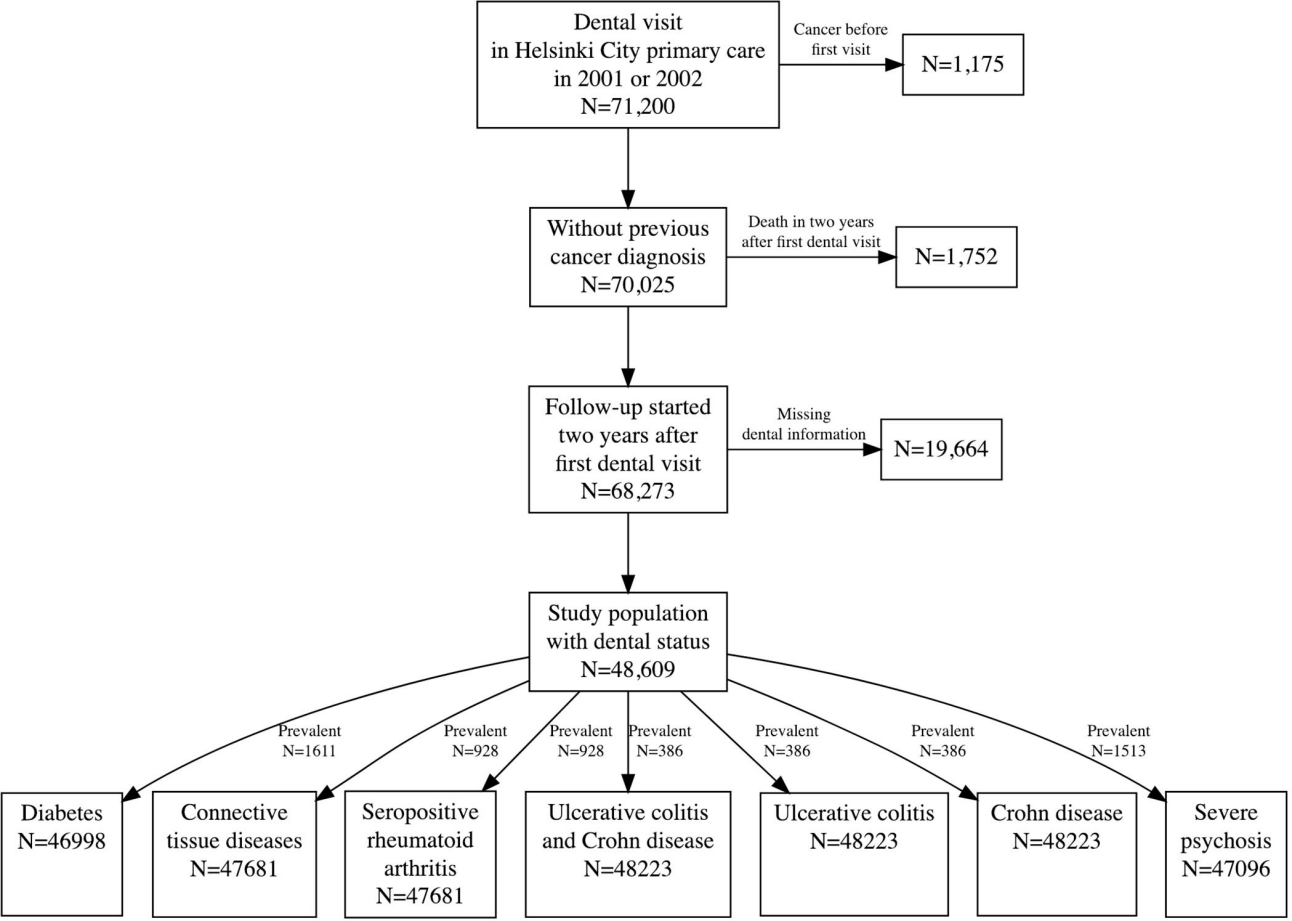
Flow chart of the study population construction.

**Figure 2:**
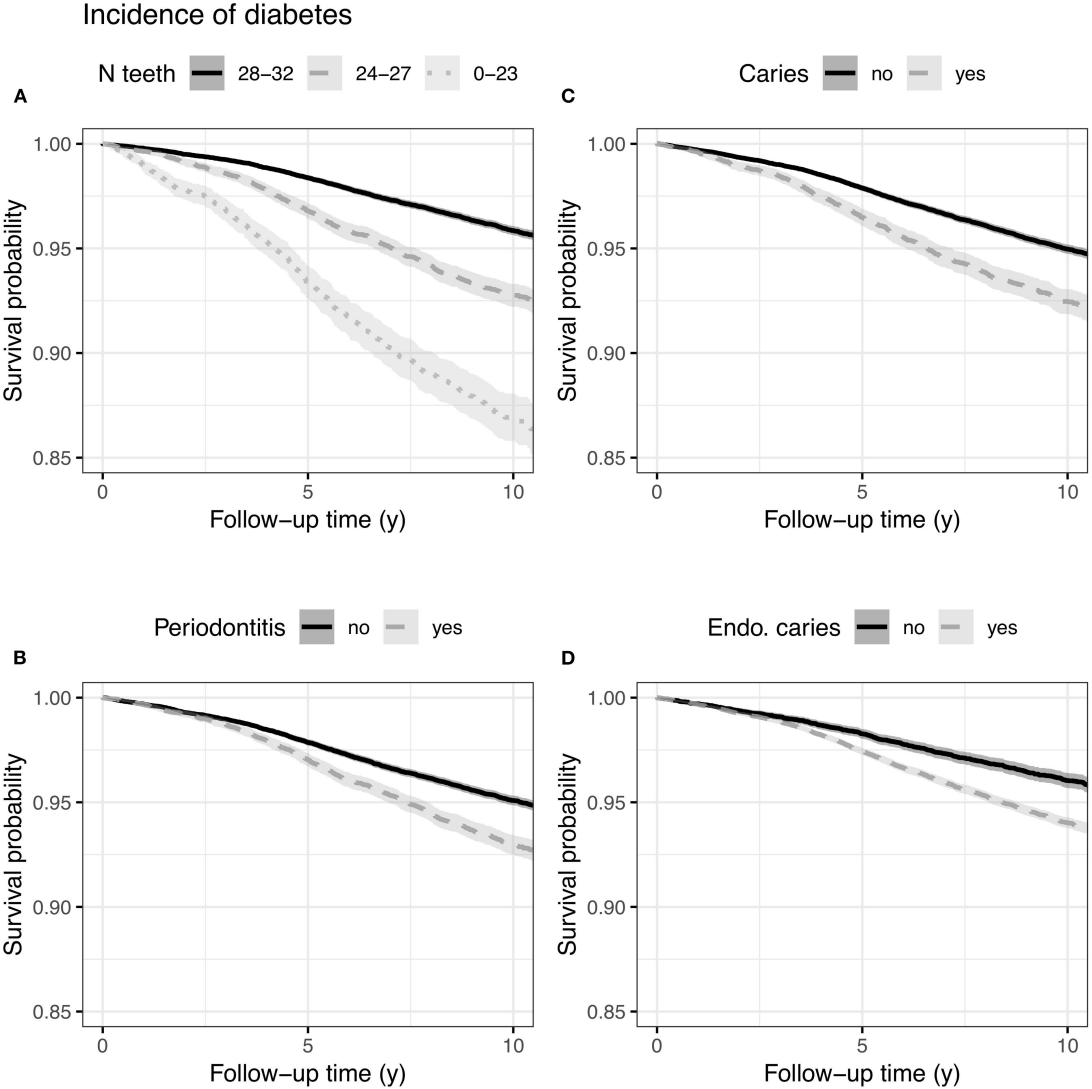
Incidence of diabetes and indicators of oral health. Kaplan–Meier curves with 95% confidence intervals. Number of teeth **(A)**, periodontitis **(B)**, caries **(C)**, endo/caries **(D)**.

The size of study cohorts varied between 46,998 for diabetes and 48,223 for IBD (ulcerative colitis and Crohn's disease; Figure 1 and Table 1). The mean follow-up time of diabetes mellitus was 9.7 years, connective tissue diseases 9.8, seropositive rheumatoid arthritis 9.9, ulcerative colitis and Crohn's disease 9.8, Crohn's disease 9.9, ulcerative colitis 9.8, and for severe psychotic and other severe mental disorders 9.8 years. The reason for different population sizes is that the prevalence of chronic conditions varies, being the highest for diabetes. About 25% of the study population had periodontitis, 17% caries, over 70% apical periodontitis, and 9% <24 teeth at the start of follow-up. The socioeconomic status of the study population and the general population of the City of Helsinki were quite similar (Supplementary Table 2). Diabetes was the only chronic condition associated with oral health variables (Table 2 and Figure 2). It turned out that having 24 to 27 teeth was associated with a higher incidence rate ratio (IRR) (1.21, 95% confidence interval 1.09–1.33) compared to having 28 or more teeth; IRR for having 23 or less was 1.40 (1.22–1.60). Having periodontitis (1.10, 1.01–1.20), caries (1.12, 1.01–1.23), or apical periodontitis lesions (1.16, 1.014–1.30) were also associated with a higher risk of diabetes (Table 2 and Supplementary Table 2). We observed a higher association between statin use and incidence of diabetes (2.49, 2.10- 2.94). There was also a relatively strong association between the number of decayed teeth (DT) and the incidence of diabetes. The IRR for DT 3–4 was 1.25 (1.10–1.42) compared to zero (Supplementary Table 3). We did not detect any other associations between oral health variables and the incidence of other chronic diseases.

**Table 1:** Baseline characteristics of the study population.

		**Outcomes**						
		**Diabetes**	**Connective tissue diseases**	**Seropositive rheumatoid arthritis**	**Ulcerative colitis or Crohn disease**	**Crohn disease**	**Ulcerative colitis**	**Severe psychosis**
All (*N*)		46,998	47,681	47,681	48,223	48,223	48,223	47,096
Age	(29, 40]	23,832 (50.7%)	23,914 (50.2%)	23,914 (50.2%)	23,951 (49.7%)	23,951 (49.7%)	23,951 (49.7%)	23,623 (50.2%)
	(40, 50]	14,892 (31.7%)	15,036 (31.5%)	15,036 (31.5%)	15,146 (31.4%)	15,146 (31.4%)	15,146 (31.4%)	14,773 (31.4%)
	(50, 60]	5,755 (12.2%)	5,969 (12.5%)	5,969 (12.5%)	6,126 (12.7%)	6,126 (12.7%)	6,126 (12.7%)	5,877 (12.5%)
	(60, 70]	1,016 (2.2%)	1,112 (2.3%)	1,112 (2.3%)	1,208 (2.5%)	1,208 (2.5%)	1,208 (2.5%)	1,124 (2.4%)
	(70, Inf]	1,503 (3.2%)	1,650 (3.5%)	1,650 (3.5%)	1,792 (3.7%)	1,792 (3.7%)	1,792 (3.7%)	1,699 (3.6%)
Gender	Male	18,531 (39.4%)	19,076 (40.0%)	19,076 (40.0%)	19,191 (39.8%)	19,191 (39.8%)	19,191 (39.8%)	18,728 (39.8%)
	Female	28,476 (60.6%)	28,605 (60.0%)	28,605 (60.0%)	29,032 (60.2%)	29,032 (60.2%)	29,032 (60.2%)	28,368 (60.2%)
Statin	No	46,144 (98.2%)	46,524 (97.6%)	46,524 (97.6%)	47,008 (97.5%)	47,008 (97.5%)	47,008 (97.5%)	45,932 (97.5%)
	Yes	854 (1.8%)	1,157 (2.4%)	1,157 (2.4%)	1,215 (2.5%)	1,215 (2.5%)	1,215 (2.5%)	1,164 (2.5%)
SES	Upper-level employees	10,086 (21.5%)	10,145 (21.3%)	10,145 (21.3%)	10,162 (21.1%)	10,162 (21.1%)	10,162 (21.1%)	10,179 (21.6%)
	Self-employed or employers	1,425 (3.0%)	1,439 (3.0%)	1,439 (3.0%)	1,444 (3.0%)	1,444 (3.0%)	1,444 (3.0%)	1,437 (3.1%)
	Lower-level employees	15,254 (32.5%)	15,315 (32.1%)	15,315 (32.1%)	15,416 (32.0%)	15,416 (32.0%)	15,416 (32.0%)	15,398 (32.7%)
	Manual workers	8,423 (17.9%)	8,547 (17.9%)	8,547 (17.9%)	8,601 (17.8%)	8,601 (17.8%)	8,601 (17.8%)	8,540 (18.1%)
	Unemployer	3,993 (8.5%)	4,087 (8.6%)	4,087 (8.6%)	4,124 (8.6%)	4,124 (8.6%)	4,124 (8.6%)	4,007 (8.5%)
	Students	1,486 (3.2%)	1,487 (3.1%)	1,487 (3.1%)	1,500 (3.1%)	1,500 (3.1%)	1,500 (3.1%)	1,439 (3.1%)
	Pensioners	3,868 (8.2%)	4,156 (8.7%)	4,156 (8.7%)	4,458 (9.2%)	4,458 (9.2%)	4,458 (9.2%)	3,640 (7.7%)
	Unknown	2,463 (5.2%)	2,505 (5.3%)	2,505 (5.3%)	2,518 (5.2%)	2,518 (5.2%)	2,518 (5.2%)	2,456 (5.2%)
No of teeth	28–32	33,515 (71.3%)	33,657 (70.6%)	33,657 (70.6%)	33,823 (70.1%)	33,823 (70.1%)	33,823 (70.1%)	33,307 (70.7%)
	24–27	9,173 (19.5%)	9,378 (19.7%)	9,378 (19.7%)	9,522 (19.7%)	9,522 (19.7%)	9,522 (19.7%)	9,267 (19.7%)
	0–23	4,319 (9.2%)	4,646 (9.7%)	4,646 (9.7%)	4,878 (10.1%)	4,878 (10.1%)	4,878 (10.1%)	4,522 (9.6%)
I index	0	12,290 (26.2%)	12,584 (26.4%)	12,584 (26.4%)	12,813 (26.6%)	12,813 (26.6%)	12,813 (26.6%)	12,430 (26.4%)
	1–2	14,455 (30.8%)	14,614 (30.6%)	14,614 (30.6%)	14,777 (30.6%)	14,777 (30.6%)	14,777 (30.6%)	14,529 (30.8%)
	3–4	8,664 (18.4%)	8,764 (18.4%)	8,764 (18.4%)	8,840 (18.3%)	8,840 (18.3%)	8,840 (18.3%)	8,655 (18.4%)
	>5	11,589 (24.7%)	11,719 (24.6%)	11,719 (24.6%)	11,793 (24.5%)	11,793 (24.5%)	11,793 (24.5%)	11,482 (24.4%)
D index	0	18,511 (39.4%)	18,621 (39.1%)	18,621 (39.1%)	18,845 (39.1%)	18,845 (39.1%)	18,845 (39.1%)	18,573 (39.4%)
	1–2	16,815 (35.8%)	16,997 (35.6%)	16,997 (35.6%)	17,191 (35.6%)	17,191 (35.6%)	17,191 (35.6%)	16,866 (35.8%)
	3–4	6,222 (13.2%)	6,351 (13.3%)	6,351 (13.3%)	6,420 (13.3%)	6,420 (13.3%)	6,420 (13.3%)	6,231 (13.2%)
	>4	5,450 (11.6%)	5,712 (12.0%)	5,712 (12.0%)	5,767 (12.0%)	5,767 (12.0%)	5,767 (12.0%)	5,426 (11.5%)
DMF index	0–13	13,401 (28.5%)	13,463 (28.2%)	13,463 (28.2%)	13,477 (27.9%)	13,477 (27.9%)	13,477 (27.9%)	13,309 (28.3%)
	14–18	11,592 (24.7%)	11,698 (24.5%)	11,698 (24.5%)	11,751 (24.4%)	11,751 (24.4%)	11,751 (24.4%)	11,583 (24.6%)
	19–23	11,168 (23.8%)	11,333 (23.8%)	11,333 (23.8%)	11,448 (23.7%)	11,448 (23.7%)	11,448 (23.7%)	11,229 (23.8%)
	>24	10,837 (23.1%)	11,187 (23.5%)	11,187 (23.5%)	11,547 (23.9%)	11,547 (23.9%)	11,547 (23.9%)	10,975 (23.3%)
CPI1	0–1	6,184 (13.2%)	6,251 (13.1%)	6,251 (13.1%)	6,322 (13.1%)	6,322 (13.1%)	6,322 (13.1%)	6,171 (13.1%)
	2	30,177 (64.2%)	30,388 (63.7%)	30,388 (63.7%)	30,676 (63.6%)	30,676 (63.6%)	30,676 (63.6%)	30,127 (64.0%)
	3–4	10,637 (22.6%)	11,042 (23.2%)	11,042 (23.2%)	11,225 (23.3%)	11,225 (23.3%)	11,225 (23.3%)	10,798 (22.9%)
Periodontitis	No	35,030 (74.5%)	35,436 (74.3%)	35,436 (74.3%)	35,817 (74.3%)	35,817 (74.3%)	35,817 (74.3%)	35,013 (74.3%)
	Yes	11,977 (25.5%)	12,245 (25.7%)	12,245 (25.7%)	12,406 (25.7%)	12,406 (25.7%)	12,406 (25.7%)	12,083 (25.7%)
Caries	No	39,165 (83.3%)	39,621 (83.1%)	39,621 (83.1%)	40,076 (83.1%)	40,076 (83.1%)	40,076 (83.1%)	39,236 (83.3%)
	Yes	7,842 (16.7%)	8,060 (16.9%)	8,060 (16.9%)	8,147 (16.9%)	8,147 (16.9%)	8,147 (16.9%)	7,860 (16.7%)
Apical periodontitis	No	12,265 (26.1%)	12,371 (25.9%)	12,371 (25.9%)	12,483 (25.9%)	12,483 (25.9%)	12,483 (25.9%)	12,249 (26.0%)
	Yes	34,733 (73.9%)	35,310 (74.1%)	35,310 (74.1%)	35,740 (74.1%)	35,740 (74.1%)	35,740 (74.1%)	34,847 (74.0%)

Population sizes for each outcome include only non-prevalent individuals. Age groups are mutually exclusive, for example (29,40], means that interval is open on the left (29 not included) and closed on the right (40 included).

**Table 2:** The number of events, event rates per 10,000 person-years with 95% confidence intervals, and unadjusted and adjusted incidence rate ratios (IRR) with 95% confidence intervals.

			**Events**	**Incidence rate** **(1/10 000)**	**IRR, univariate**	**IRR, adjusted**
Diabetes	N. teeth	28–32	1,412	42.37 (40.19, 44.64)	(Reference)	(Reference)
		24–27	650	74.17 (68.57, 80.09)	1.75 (1.60, 1.92)	1.21 (1.09, 1.33)
		0–23	471	139.88 (127.53, 153.10)	3.30 (2.97, 3.66)	1.40 (1.22, 1.60)
	Periodontitis	No	1,702	50.06 (47.71, 52.50)	(Reference)	(Reference)
		Yes	831	72.55 (67.70, 77.65)	1.45 (1.33, 1.57)	1.10 (1.01, 1.20)
	Caries	No	1,949	51.39 (49.13, 53.72)	(Reference)	(Reference)
		Yes	584	77.60 (71.44, 84.16)	1.51 (1.38, 1.66)	1.12 (1.01, 1.23)
	Apical periodontitis	No	478	40.48 (36.93, 44.28)	(Reference)	(Reference)
		Yes	2,055	61.09 (58.48, 63.79)	1.51 (1.37, 1.67)	1.16 (1.04, 1.30)
Connective tissue diseases	N. teeth	28–32	338	9.97 (8.94, 11.10)	(Reference)	(Reference)
		24–27	129	14.07 (11.75, 16.72)	1.41 (1.15, 1.73)	1.21 (0.97, 1.50)
		0–23	43	11.36 (8.22, 15.30)	1.14 (0.83, 1.56)	1.00 (0.68, 1.47)
	Periodontitis	No	372	10.67 (9.61, 11.81)	(Reference)	(Reference)
		Yes	138	11.52 (9.68, 13.61)	1.08 (0.89, 1.31)	1.09 (0.88, 1.34)
	Caries	No	419	10.77 (9.76, 11.85)	(Reference)	(Reference)
		Yes	91	11.48 (9.24, 14.09)	1.07 (0.85, 1.34)	0.97 (0.76, 1.24)
	Apical periodontitis	No	119	9.89 (8.19, 11.83)	(Reference)	(Reference)
		Yes	391	11.23 (10.15, 12.40)	1.14 (0.93, 1.40)	0.93 (0.73, 1.18)
Seropositive rheumatoid arthritis	N. teeth	28–32	67	1.97 (1.53, 2.50)	(Reference)	(Reference)
		24–27	29	3.15 (2.11, 4.52)	1.60 (1.03, 2.47)	1.30 (0.81, 2.07)
		0–23	16	4.21 (2.41, 6.84)	2.14 (1.24, 3.69)	1.78 (0.89, 3.57)
	Periodontitis	No	81	2.31 (1.84, 2.88)	(Reference)	(Reference)
		Yes	31	2.58 (1.75, 3.66)	1.11 (0.74, 1.68)	1.03 (0.66, 1.59)
	Caries	No	91	2.33 (1.88, 2.86)	(Reference)	(Reference)
		Yes	21	2.64 (1.63, 4.03)	1.13 (0.70, 1.82)	1.01 (0.61, 1.68)
	Apical periodontitis	No	29	2.40 (1.61, 3.45)	(Reference)	(Reference)
		Yes	83	2.37 (1.89, 2.94)	0.99 (0.65, 1.51)	0.77 (0.47, 1.27)
Ulcerative colitis and Crohn disease	N. teeth	28–32	141	4.13 (3.48, 4.87)	(Reference)	(Reference)
		24–27	36	3.85 (2.70, 5.33)	0.93 (0.65, 1.35)	1.18 (0.80, 1.74)
		0–23	11	2.76 (1.38, 4.94)	0.67 (0.36, 1.23)	1.35 (0.63, 2.90)
	Periodontitis	No	137	3.88 (3.26, 4.59)	(Reference)	(Reference)
		Yes	51	4.19 (3.12, 5.51)	1.08 (0.78, 1.49)	1.36 (0.97, 1.91)
	Caries	No	159	4.03 (3.43, 4.71)	(Reference)	(reference)
		Yes	29	3.61 (2.41, 5.18)	0.89 (0.60, 1.33)	0.95 (0.62, 1.45)
	Apical periodontitis	No	52	4.27 (3.19, 5.60)	(Reference)	(reference)
		Yes	136	3.85 (3.23, 4.56)	0.90 (0.65, 1.24)	0.89 (0.61, 1.31)
Crohn disease	N. teeth	28–32	28	0.82 (0.54, 1.18)	(Reference)	(Reference)
		24–27	9	0.96 (0.44, 1.83)	1.17 (0.55, 2.49)	1.52 (0.68, 3.42)
		0–23	1	0.25 (0.01, 1.40)	0.31 (0.04, 2.25)	0.59 (0.06, 5.32)
	Periodontitis	No	29	0.82 (0.55, 1.18)	(Reference)	(reference)
		Yes	9	0.74 (0.34, 1.40)	0.90 (0.43, 1.90)	1.12 (0.51, 2.45)
	Caries	No	34	0.86 (0.60, 1.20)	(Reference)	(reference)
		Yes	4	0.50 (0.14, 1.27)	0.58 (0.20, 1.63)	0.57 (0.19, 1.71)
	Apical periodontitis	No	14	1.15 (0.63, 1.93)	(Reference)	(reference)
		Yes	24	0.68 (0.43, 1.01)	0.59 (0.31, 1.14)	0.71 (0.32, 1.57)
Ulcerative colitis	N. teeth	28–32	113	3.31 (2.73, 3.98)	(Reference)	(reference)
		24–27	27	2.89 (1.90, 4.20)	0.87 (0.57, 1.33)	1.10 (0.70, 1.71)
		0–23	10	2.51 (1.20, 4.61)	0.76 (0.40, 1.45)	1.53 (0.67, 3.48)
	Periodontitis	No	108	3.06 (2.51, 3.69)	(Reference)	(reference)
		Yes	42	3.45 (2.49, 4.67)	1.13 (0.79, 1.61)	1.42 (0.98, 2.07)
	Caries	No	125	3.17 (2.64, 3.78)	(Reference)	(reference)
		Yes	25	3.11 (2.01, 4.59)	0.98 (0.64, 1.51)	1.06 (0.67, 1.67)
	Endo. caries	No	38	3.12 (2.21, 4.28)	(Reference)	(reference)
		Yes	112	3.17 (2.61, 3.82)	1.02 (0.70, 1.47)	0.96 (0.62, 1.49)
Severe psychosis	N. teeth	28–32	28	0.82 (0.54, 1.18)	(Reference)	(reference)
		24–27	9	0.96 (0.44, 1.83)	1.17 (0.55, 2.49)	1.15 (0.93, 1.41)
		0–23	1	0.25 (0.01, 1.40)	0.31 (0.04, 2.25)	0.86 (0.60, 1.21)
	Periodontitis	No	29	0.82 (0.55, 1.18)	(Reference)	(reference)
		Yes	9	0.74 (0.34, 1.40)	0.90 (0.43, 1.90)	1.04 (0.86, 1.25)
	Caries	No	34	0.86 (0.60, 1.20)	(Reference)	(reference)
		Yes	4	0.50 (0.14, 1.27)	0.58 (0.20, 1.63)	1.12 (0.91, 1.38)
	Apical periodontitis	No	14	1.15 (0.63, 1.93)	(Reference)	(reference)
		Yes	24	0.68 (0.43, 1.01)	0.59 (0.31, 1.14)	1.19 (0.95, 1.48)

Adjusted using Poisson regression for sex, age, socioeconomic status, usage of statins in baseline, i-index, D-index, and CPI.

We calculated the number needed to harm (NNH) for new-onset diabetes in 1 year for three oral health variables (Table 3). NNH for PD was 1,736 (95% confidence interval 903–22,103), for caries 1,342 (709, 12,497), and NNH for having <24 teeth compared to having 28 or more teeth was 262 (190–420).

**Table 3:** Incidence of diabetes, number needed to harm (NNH) based on the additive Poisson regression model.

		**NNH (95% confidence interval)**
N. teeth	28–32	(Reference)
	24–27	1,004 (625, 2,561)
	0–23	262 (190, 420)
Periodontitis	No	(Reference)
	Yes	1,736 (903, 22,103)
Caries	No	(Reference)
	Yes	1,342 (709, 12,497)
Apical periodontitis	No	(Reference)
	Yes	2,052 (1,029, 349,261)

Adjusted for gender, age, socioeconomic status, usage of statins in baseline, i-index, D-index, and CPI.

## Discussion

We collected these from the largely presentative population (N = 68,273) observational register study with a long follow-up (10 years). The incidence is measured by the initiation of drug treatment for specific conditions verified by special reimbursement. Our main finding was that oral health indices were related to diabetes but not to other chronic conditions. Thus, our findings support the close association of oral health, especially PD, to metabolic deterioration of glucose metabolism.

### Strengths and limitations of this study

The study population consisted of patients with at least one visit to dental healthcare in the City of Helsinki in 2 years period. This means that individuals without any dental visits were not eligible for the study. In principle, this non-eligible population includes people without any dental visits and those utilizing only the private sector dental healthcare. Usage of private healthcare may have caused some selection of the study population because about 36% of dental care was covered by it in the study period. As private care is most likely more commonly used by higher socioeconomic groups, this could imply that the selection process is drifted to lower socioeconomic groups. Still, as we had access to SES, we could also verify that higher SES were using the public health sector to a large extent (Table 1 and Supplementary Table 2). Among the potential confounders in this study were socio-demographic characteristics, such as age, sex, and SES status, available for the entire study population. The study was limited by the lack of information on smoking, alcohol use, and dietary habits, which may confound the findings because both are known risk factors for chronic diseases [30]. Our predecessor article found a strong positive association between periodontitis at baseline and subsequent risk of fatal pancreatic cancer. However, at the same time, we did not detect any association between periodontitis and lung cancer, which may be interpreted to indicate that confounding by smoking is probably not strong [32]. Altogether, additional studies are needed with more detailed measurements of confounders such as smoking and alcohol use to confirm these results. The diabetes diagnosis was based on the information reimbursement for the drug treatment for diabetes. This covers in practice all the subjects with drug treatment and the practice is to start medication already at the time of diagnosis [33]. However, it does not include the subjects with undiagnosed diabetes and asymptomatic hyperglycemia, and unfortunately HbA1c level information is not available from these registers. This is a weakness of the study, but if anything these shortcomings are likely to weaken the associations found in this study.

We also addressed statin medication, which certain but few studies have revealed with a low increased risk for the development of diabetes. Still, the risk is low both in absolute terms and when related to the reduction in coronary events. Clinical practice in patients with moderate or high cardiovascular risk or existing cardiovascular disease is well-documented [34], while statins are known to benefit the treatment and course of chronic periodontitis. Apart from their established LDL-cholesterol lowering effects, statins have shown additional secondary effects, including anti-inflammatory, immunomodulatory, antioxidant, antithrombotic, and endothelium stabilization effects, and promote angiogenesis [35]. Recent retrospective studies have demonstrated that patients with chronic periodontitis treated with simvastatin or atorvastatin had lower indexes of periodontitis than those not receiving statins [36, 37]. Atorvastatin can also restore endothelium-dependent vasodilation in normocholesterolemic cigarette smokers independent of changes in the lipids [38, 39]. Statins have recently been recorded to have beneficial effects on chronic periodontitis among smokers [40]. In our study, people with statin therapy were at higher risk of diabetes with IRR 2.49 (2.10-2.94). However, this number should be interpreted with great caution because it may contain the so-called “table two” fallacy. Table two fallacy is present when effects other than primary exposure are interpreted [41]. The main potential confounding factors between oral chronic infections and diabetes are tobacco, alcohol, socioeconomic status, age and sex, and genetic and dietary factors. Among the potential confounders in this study were socio-demographic characteristics, such as age, sex, and socio-economic status, available for the entire study population.

We measured our outcomes as starting of new special reimbursement. This means that the diagnosis of the condition has taken place earlier. This lag may vary between the diagnosis and between individuals considerably. This means that results may be biased if the lag between diagnosis and the start of reimbursement is affected by oral health status. We tried to control this source of bias by starting a follow-up after 2 years of the first dental visit recorded. We could assess the type of diabetes only by the kind of therapy. However, those treated only with insulin comprise type 1 diabetic patients and some long-standing type 2 diabetic patients whose disease can be classified as insulin-requiring. The demarcation between specific types of new-onset diabetes in adults in individual cases may also be somewhat arbitrary in clinical practice. It is also possible regarding diabetic subjects treated with lifestyle only. However, as lifestyle interventions are difficult to implement and may delay unnecessarily the start of drug treatment, the Finnish Current Care guideline (original version published in 2007 and updated several times) recommended that drug treatment with metformin should be initiated if not contraindicated concomitantly with lifestyle interventions [42]. We have previously shown that the implementation of these guidelines has been successful, [43] and thus, nearly all the subjects with a clinically verified diagnosis of diabetes were included making this population representative.

The main strength of the exposure measurements is that they contained detailed clinical information about oral health and dental procedures. Our primary exposures—periodontal, cariological status, and apical periodontitis—were determined by procedure codes, which means that the number of false positives is very low. Studies investigating the association between periodontal disease and diabetes and other chronic diseases have used various measures to define the periodontal disease and how disease progression is ascertained. There is no standardized definition or clinical criteria for periodontal disease in periodontal epidemiological research, hindering comparisons of studies examining the association between periodontal disease and chronic diseases [44]. Periodontal disease is generally diagnosed by probing and measuring alveolar bone height with radiographs. In this study, we used the information on dental status presented by the number of teeth. Dental infections, caries, or periodontitis can potentially be assumed as the reason for the extracted teeth. Earlier studies have hypothesized that missing teeth reflect an individual lifetime accumulation of oral inflammation. In the national FINRISK 1997 study Finnish population-based survey of 8,446 subjects with 13 years of follow-up, Liljestrand et al. revealed that missing teeth predict incident cardiovascular events, diabetes, and death, and periodontitis was the main cause of tooth loss in the middle-aged and elderly [45]. While, other studies like Chauncey et al. and Jovino-Silvera et al. showed caries complications to be the primary reason for tooth extraction. In these studies, the size of the studied population is less than one thousand [46, 47]. Caries can lead to dental pulp necrosis with subsequent infection spread in the apical area and beyond. It can leave chronic inflammation to persist in the apical area, apical periodontitis [48].

In this study, we used the division into three teeth groups due to a lack of consensus. The groups with the number of teeth overlap with each other concerning the evident causes due to which individual teeth are extracted, but hypothetical cause grouping may aid in the interpretation of results: the first group with teeth 28 to 32 represent periodontally healthy subjects or lack of or extractions of third molars; those with teeth 24 to 27 may have additionally lost them due to orthodontic reasons, periodontitis, or caries; and those with teeth 0 to 23 most likely suffer from chronic periodontitis [45]. Thus, these groups with fewer teeth eventually represent ongoing or treated advanced oral disease with a plausible systemic inflammatory burden.

We used reported history of procedure codes and dental status represented by the number of teeth, oral health indices, initial caries, decayed/missing/filled teeth, and need for periodontal treatment according to the involvement of gingival pockets. Furthermore, we defined periodontitis as a binary variable (no/yes) based on the procedure codes of periodontitis treatment in the years 2001 and 2002, when we collected data on patients' oral health status. Overall, the collected data support the association between periodontitis, caries, apical periodontitis, and diabetes.

### Comparisons with other studies

Many previous studies investigated biological connections between periodontitis and diabetes-focused on the impact of diabetes on periodontal pathogenesis. There is evidence for the bidirectional connection between these two diseases with associated feedback effects. A dysregulated immune system is essential to the pathogenesis of diabetes and its complications. Systemic changes in cytokine and matrix metalloproteinase (MMP) levels impact the pathogenesis of type 2 diabetes, associated with physiological, nutritional, and metabolic changes, including hyperglycemia, production of advanced glycation end-products (AGE), hyperlipidemia, and increased adiposity [44, 49]. These mechanisms can affect by weakening the individual's immune response and periodontal condition. Proteins are glycated and eventually converted to AGE products in persistent hyperglycemia. These irreversible glycation processes of proteins have several consequences, including immune and proinflammatory dysregulation manifested by a pronounced, long-lasting inflammatory state and weakened self-limitation resolution of immune responses [44, 49]. These processes mediate pathophysiological mechanisms promoting the development and progression of periodontitis in diabetes, interfering with the physiologic tissue repair and wound healing. When AGE products bind to signaling receptors of several cell types, one of the results is the production and release of reactive oxygen species, proinflammatory mediators, and MMPs. The reactive oxygen species, cytokines, and proteases promote inflammation and ultimately exacerbate periodontal tissue destruction through an exaggerated inflammatory response and limited tissue repair [50]. Concerning the potential impact of periodontitis on the disease processes of diabetes, there is little biological evidence available. The periodontal microbiota appears unaltered by diabetes, and there is little evidence that it may influence glycemic control. The systemic inflammation triggered by periodontitis can affect the regulation of the serum glucose level through an increase in the levels of inflammatory mediators, such as tumor necrosis factor-alpha and interleukin-6, MMPs, oxygen radicals, and acute-phase proteins, which interfere with the glucose control mechanism, inhibit and inactivate the insulin receptors, and reduce the uptake of glucose into the cell. In the presence of severe periodontitis, serum glucose levels can become elevated over the years in a clinically significant manner, even without diabetes. MMP-8 can proteolytically process insulin receptors [11]. If diabetes is already present with the simultaneous presence of untreated severe periodontitis, proper glycemic control is probably more challenging, and the risk for diabetes complications is increased [51].

Some former studies have found that the risk of systemic disease can be decreased with periodontal therapy. The various clinical trials demonstrated that periodontal treatment prevented or modified the progression of systemic diseases. All of the studies conducted were limited by a small sample size and inconsistent outcome measures across studies, and the limited duration of follow-up [52, 53]. In Sabharwal et al.'s review article, the majority of 23 randomized clinical trials revealed consistent and moderate effects of periodontal treatment on serum glycemic control in type 2 diabetic individuals. The treatment of periodontitis may thus contribute to improvements in the mouth and throughout the body, with a reduction of the concentrations of inflammatory mediators and MMPs in the blood resulting in the reduction of the average serum glucose levels and improvements in the demonstrated lipid profiles, in general, improving the control of diabetes [52]. A recent study by D'Aiuto et al. showed that periodontitis treatment reduced 0.6% HbA1c in patients (*N* = 264) with type 2 diabetes and moderate-to-severe periodontitis after 12 months [13].

This study comprises a large unselected population with representative cohorts of patients with periodontitis and apical periodontitis followed up over 10 years. Despite the limitations inherent in this type of study, the results are likely to be generalizable to similar populations of individuals with chronic oral diseases. The association exists between chronic oral diseases and diabetes, which warrants close collaboration among each patient's healthcare professionals, especially among medical and dental care providers.

## Data availability statement

The original contributions presented in the study are included in the article/Supplementary material, further inquiries can be directed to the corresponding author/s.

## Ethics statement

Written informed consent was not required for this study in accordance with the local legislation and institutional requirements.

## Author contributions

All authors participated in the data interpretation and the manuscript's critical revision. All authors exerted full access to all data (including programming code, statistical reports, and tables) during the study and are responsible for the data integrity and data analysis accuracy. All authors contributed to the article and approved the submitted version.

## Funding

The work was supported by grants from the Finnish Dental Association, Finnish Outpatient Research Foundation, the University of Helsinki Tulevaisuusrahasto, the Helsinki University Hospital Research Foundation, the Apollonia Foundation Helsinki, Finland, and Karolinska Institutet, Stockholm, Sweden. The funders exerted no roles in study design, data collection analysis, interpretation, or report.

## Conflict of interest

The authors declare that the research was conducted in the absence of any commercial or financial relationships that could be construed as a potential conflict of interest.

## Publisher's note

All claims expressed in this article are solely those of the authors and do not necessarily represent those of their affiliated organizations, or those of the publisher, the editors and the reviewers. Any product that may be evaluated in this article, or claim that may be made by its manufacturer, is not guaranteed or endorsed by the publisher.

## Abbreviations

AGE: advanced glycation end-products
AP: periodontitis
CPI: periodontal treatment need index
DMTF: decayed/missing/filled teeth
DT: decayed teeth
FCR: Finnish Cancer Registry
IBD: inflammatory bowel disease
ICD-10: International Classification of Diseases
I: primary caries
IRR: incidence rate ratios
MMP: matrix metalloproteinases
NNH: number needed to harm
SII: Finnish Social Insurance Institution
SES: socioeconomic status.
