# Supplementary material for: Oral health associated with incident diabetes but not other chronic diseases: A register-based cohort study
Source: Front Oral Health. 2022 Aug 18;3:956072. doi: 10.3389/froh.2022.956072 (PMC9433743; doi:10.3389/froh.2022.956072)
Supplement: Supplementary file 1 [file Table_1.DOCX]

Supplement Table 1. Social Insurance Institution special reimbursement codes and ICD10 codes included.

| SII code | ICD10 code |
| --- | --- |
| Diabetes (103) | E10, E101, E102, E103, E104, E109, E11, E110, E111, E112, E113, E114, E115, E116, E117, E118, E119, E13, E131, E132, E134, E138, E139, E14, E141, E146, E149, E891 |
| Connective tissue diseases (202) | A046, H201, H30, I330, J84, J841, K509, K519, K732, K743, K830, L405, M02, M021, M028, M029, M05, M050, M058, M059, M06, M060, M061, M064, M069, M07, M08, M084, M139, M30, M31, M310, M313, M315, M316, M317, M318, M32, M321, M329, M33, M331, M332, M339, M34, M341, M35, M350, M351, M352, M353, M355, M359, M45, M450, M461, M469, M941, N03 |
| Ulcerative  colitis and Crohn’s disease (208) | K50, K500, K501, K508, K509, K51, K510, K511, K512, K513, K515, K518, K519 |
| Severe psychotic and other severe mental  disorders (112) | F01, F018, F019, F03, F20, F200, F201, F202, F203, F205, F206, F208, F209, F21, F22, F220, F223, F228, F229, F23, F230, F231, F232, F233, F239, F24, F25, F250, F251, F252, F258, F259, F28, F29, F290, F299, F301, F302, F31, F310, F311, F312, F313, F314, F315, F316, F317, F318, F319, F32, F323, F333, F61, F84, F840, F841, F845, G20, G300, G301, G308, G309, G310, G409 |

Supplement Table 2. Sosioeconomic status in City of Helsinki on year 2000.

| Sosioeconomic status | N | Percentage |
| --- | --- | --- |
| Self-employed or employers | 19959 | 3.6 % |
| Upper-level employees | 107627 | 19.4 % |
| Lower-level employees | 134477 | 24.2 % |
| Manual workers | 90422 | 16.3 % |
| Students | 35830 | 6.5 % |
| Pensioners | 104886 | 18.9 % |
| Other (includes unemployed) | 31924 | 5.7 % |
| Unknown | 30349 | 5.5 % |
| All | 555474 | 100.0 % |

Supplement Table 3. Oral health and chronic disease incidence/ RR from Poisson regression models.

| **RR, adjusted by Poisson regression full model**  Key to variable names and categories:   - sukup (sex) : ‘male’,‘female’ - Relevel(tage, ref = 3) (age) : ‘(50,60]’,‘(29,40]’,‘(40,50]’,‘(60,70]’,‘(70,Inf]’ - t1sose_2000.f (sosio-economic position): ‘Upper-level employees’,‘Self-employed or employers’,‘Lower-level employees’,‘Manual workers’,‘Unemployer’,‘Students’,‘Pensioners’,‘Unknown’ - statin.presc.bl (baseline statin use) : ‘no’,‘yes’ - cN.teeth.1 (number of teeth): ‘28-32’,‘24-27’,‘0-23’ - i.index.max (i index): ‘0’,‘1-2’,‘3-4’,‘>5’ - D.index.max (D index): ‘0’,‘1-2’,‘3-4’,‘>4’ - Relevel(DMF.index.max, ref = 3) (DMF index): ‘19-23’,‘0-13’,‘14-18’,‘>24’ - cpi1.max (CPI1 index): ‘0-1’,‘2’,‘3-4’ - periodontitis : ‘No’,‘Yes’ - caries : ‘No’,‘Yes’ - endoCaries (Apical periodontitis) : ‘No’,‘Yes’   *Diabetes*   \|  \| RR \| 2.5% \| 97.5% \| P \| \| --- \| --- \| --- \| --- \| --- \| \| sukupfemale \| 0.8349 \| 0.7691 \| 0.9063 \| 0.000016 \| \| Relevel(tage, ref = 3)(29,40] \| 0.3846 \| 0.3388 \| 0.4367 \| 0.000000 \| \| Relevel(tage, ref = 3)(40,50] \| 0.6547 \| 0.5874 \| 0.7296 \| 0.000000 \| \| Relevel(tage, ref = 3)(60,70] \| 0.9067 \| 0.7459 \| 1.1020 \| 0.325000 \| \| Relevel(tage, ref = 3)(70,Inf] \| 0.4425 \| 0.3471 \| 0.5641 \| 0.000000 \| \| t1sose_2000.fSelf-employed or employers \| 1.3136 \| 1.0052 \| 1.7166 \| 0.045730 \| \| t1sose_2000.fLower-level employees \| 1.4921 \| 1.3009 \| 1.7114 \| 0.000000 \| \| t1sose_2000.fManual workers \| 1.7912 \| 1.5511 \| 2.0686 \| 0.000000 \| \| t1sose_2000.fUnemployer \| 1.8539 \| 1.5708 \| 2.1881 \| 0.000000 \| \| t1sose_2000.fStudents \| 1.7458 \| 1.3484 \| 2.2603 \| 0.000024 \| \| t1sose_2000.fPensioners \| 2.4732 \| 2.0800 \| 2.9408 \| 0.000000 \| \| t1sose_2000.fUnknown \| 1.7859 \| 1.4639 \| 2.1787 \| 0.000000 \| \| statin.presc.blyes \| 2.4861 \| 2.1045 \| 2.9369 \| 0.000000 \| \| cN.teeth.124-27 \| 1.2062 \| 1.0900 \| 1.3349 \| 0.000288 \| \| cN.teeth.10-23 \| 1.3976 \| 1.2196 \| 1.6016 \| 0.000001 \| \| i.index.max1-2 \| 0.9041 \| 0.8163 \| 1.0013 \| 0.052940 \| \| i.index.max3-4 \| 1.0389 \| 0.9239 \| 1.1682 \| 0.523973 \| \| i.index.max>5 \| 0.8910 \| 0.7907 \| 1.0041 \| 0.058358 \| \| D.index.max1-2 \| 1.0607 \| 0.9551 \| 1.1779 \| 0.270778 \| \| D.index.max3-4 \| 1.2483 \| 1.0968 \| 1.4208 \| 0.000783 \| \| D.index.max>4 \| 1.2807 \| 1.1200 \| 1.4644 \| 0.000297 \| \| Relevel(DMF.index.max, ref = 3)0-13 \| 0.9059 \| 0.7929 \| 1.0349 \| 0.145624 \| \| Relevel(DMF.index.max, ref = 3)14-18 \| 1.0355 \| 0.9206 \| 1.1647 \| 0.560967 \| \| Relevel(DMF.index.max, ref = 3)>24 \| 1.0604 \| 0.9506 \| 1.1830 \| 0.293007 \| \| cpi1.max2 \| 1.2314 \| 1.0679 \| 1.4198 \| 0.004180 \| \| cpi1.max3-4 \| 1.3383 \| 1.1462 \| 1.5625 \| 0.000228 \| \| periodontitisYes \| 1.1029 \| 1.0102 \| 1.2042 \| 0.028813 \| \| cariesYes \| 1.1188 \| 1.0139 \| 1.2347 \| 0.025511 \| \| endoCariesYes \| 1.1644 \| 1.0396 \| 1.3042 \| 0.008521 \|   *Connective tissue diseases*   \|  \| RR \| 2.5% \| 97.5% \| P \| \| --- \| --- \| --- \| --- \| --- \| \| sukupfemale \| 1.7502 \| 1.4297 \| 2.1426 \| 0.000000 \| \| Relevel(tage, ref = 3)(29,40] \| 0.6670 \| 0.4986 \| 0.8922 \| 0.006356 \| \| Relevel(tage, ref = 3)(40,50] \| 0.8199 \| 0.6255 \| 1.0748 \| 0.150498 \| \| Relevel(tage, ref = 3)(60,70] \| 0.5570 \| 0.2665 \| 1.1643 \| 0.119825 \| \| Relevel(tage, ref = 3)(70,Inf] \| 0.6319 \| 0.2741 \| 1.4570 \| 0.281536 \| \| t1sose_2000.fSelf-employed or employers \| 0.8227 \| 0.4270 \| 1.5850 \| 0.559661 \| \| t1sose_2000.fLower-level employees \| 1.3017 \| 1.0081 \| 1.6809 \| 0.043193 \| \| t1sose_2000.fManual workers \| 1.5932 \| 1.1972 \| 2.1203 \| 0.001403 \| \| t1sose_2000.fUnemployer \| 1.1157 \| 0.7616 \| 1.6344 \| 0.574025 \| \| t1sose_2000.fStudents \| 1.0030 \| 0.5594 \| 1.7984 \| 0.991880 \| \| t1sose_2000.fPensioners \| 0.9296 \| 0.5617 \| 1.5383 \| 0.776239 \| \| t1sose_2000.fUnknown \| 1.1267 \| 0.7200 \| 1.7632 \| 0.601522 \| \| statin.presc.blyes \| 1.3139 \| 0.7558 \| 2.2841 \| 0.333281 \| \| cN.teeth.124-27 \| 1.2083 \| 0.9711 \| 1.5035 \| 0.089762 \| \| cN.teeth.10-23 \| 1.0001 \| 0.6805 \| 1.4700 \| 0.999502 \| \| i.index.max1-2 \| 0.9211 \| 0.7310 \| 1.1607 \| 0.485871 \| \| i.index.max3-4 \| 0.8643 \| 0.6575 \| 1.1361 \| 0.295851 \| \| i.index.max>5 \| 0.9127 \| 0.7054 \| 1.1810 \| 0.487169 \| \| D.index.max1-2 \| 1.2573 \| 1.0029 \| 1.5762 \| 0.047135 \| \| D.index.max3-4 \| 1.1254 \| 0.8264 \| 1.5326 \| 0.453440 \| \| D.index.max>4 \| 1.3145 \| 0.9497 \| 1.8194 \| 0.099207 \| \| Relevel(DMF.index.max, ref = 3)0-13 \| 0.7045 \| 0.5367 \| 0.9248 \| 0.011628 \| \| Relevel(DMF.index.max, ref = 3)14-18 \| 0.8459 \| 0.6618 \| 1.0813 \| 0.181464 \| \| Relevel(DMF.index.max, ref = 3)>24 \| 0.9023 \| 0.7008 \| 1.1618 \| 0.425473 \| \| cpi1.max2 \| 0.8877 \| 0.6839 \| 1.1521 \| 0.370410 \| \| cpi1.max3-4 \| 0.7329 \| 0.5315 \| 1.0105 \| 0.057933 \| \| periodontitisYes \| 1.0868 \| 0.8838 \| 1.3363 \| 0.430273 \| \| cariesYes \| 0.9709 \| 0.7631 \| 1.2352 \| 0.809955 \| \| endoCariesYes \| 0.9265 \| 0.7291 \| 1.1775 \| 0.532626 \|   *Seropositive rheumatoid arthritis*   \|  \| RR \| 2.5% \| 97.5% \| P \| \| --- \| --- \| --- \| --- \| --- \| \| sukupfemale \| 2.4865 \| 1.5671 \| 3.9454 \| 0.000110 \| \| Relevel(tage, ref = 3)(29,40] \| 0.5521 \| 0.3055 \| 0.9977 \| 0.049108 \| \| Relevel(tage, ref = 3)(40,50] \| 0.6535 \| 0.3798 \| 1.1245 \| 0.124455 \| \| Relevel(tage, ref = 3)(60,70] \| 0.8117 \| 0.2726 \| 2.4174 \| 0.707898 \| \| Relevel(tage, ref = 3)(70,Inf] \| 0.1769 \| 0.0215 \| 1.4565 \| 0.107317 \| \| t1sose_2000.fSelf-employed or employers \| 1.3707 \| 0.4666 \| 4.0270 \| 0.566289 \| \| t1sose_2000.fLower-level employees \| 1.0454 \| 0.6063 \| 1.8027 \| 0.873000 \| \| t1sose_2000.fManual workers \| 1.2988 \| 0.7014 \| 2.4050 \| 0.405540 \| \| t1sose_2000.fUnemployer \| 0.7328 \| 0.3044 \| 1.7639 \| 0.487884 \| \| t1sose_2000.fStudents \| 0.9661 \| 0.2864 \| 3.2590 \| 0.955696 \| \| t1sose_2000.fPensioners \| 1.0995 \| 0.4518 \| 2.6758 \| 0.834430 \| \| t1sose_2000.fUnknown \| 1.2272 \| 0.5141 \| 2.9299 \| 0.644640 \| \| statin.presc.blyes \| 1.0144 \| 0.3094 \| 3.3252 \| 0.981225 \| \| cN.teeth.124-27 \| 1.2988 \| 0.8134 \| 2.0738 \| 0.273507 \| \| cN.teeth.10-23 \| 1.7840 \| 0.8913 \| 3.5710 \| 0.102065 \| \| i.index.max1-2 \| 1.2656 \| 0.7739 \| 2.0698 \| 0.347861 \| \| i.index.max3-4 \| 0.8311 \| 0.4371 \| 1.5802 \| 0.572487 \| \| i.index.max>5 \| 1.2342 \| 0.7064 \| 2.1564 \| 0.459763 \| \| D.index.max1-2 \| 1.1582 \| 0.7105 \| 1.8882 \| 0.555791 \| \| D.index.max3-4 \| 1.1290 \| 0.5890 \| 2.1643 \| 0.714736 \| \| D.index.max>4 \| 1.3748 \| 0.7052 \| 2.6804 \| 0.350011 \| \| Relevel(DMF.index.max, ref = 3)0-13 \| 0.5639 \| 0.3085 \| 1.0308 \| 0.062696 \| \| Relevel(DMF.index.max, ref = 3)14-18 \| 0.7139 \| 0.4195 \| 1.2150 \| 0.214176 \| \| Relevel(DMF.index.max, ref = 3)>24 \| 0.7590 \| 0.4503 \| 1.2796 \| 0.300794 \| \| cpi1.max2 \| 0.9697 \| 0.5392 \| 1.7437 \| 0.918087 \| \| cpi1.max3-4 \| 1.0348 \| 0.5244 \| 2.0418 \| 0.921442 \| \| periodontitisYes \| 1.0273 \| 0.6625 \| 1.5931 \| 0.904217 \| \| cariesYes \| 1.0124 \| 0.6096 \| 1.6812 \| 0.962073 \| \| endoCariesYes \| 0.7730 \| 0.4704 \| 1.2702 \| 0.309621 \|   *Ulcerative colitis or Crohn disease*   \|  \| RR \| 2.5% \| 97.5% \| P \| \| --- \| --- \| --- \| --- \| --- \| \| sukupfemale \| 0.7315 \| 0.5410 \| 0.9890 \| 0.042163 \| \| Relevel(tage, ref = 3)(29,40] \| 2.1735 \| 1.0825 \| 4.3640 \| 0.029048 \| \| Relevel(tage, ref = 3)(40,50] \| 1.7989 \| 0.8989 \| 3.6001 \| 0.097174 \| \| Relevel(tage, ref = 3)(60,70] \| 4.2341 \| 1.4944 \| 11.9966 \| 0.006609 \| \| Relevel(tage, ref = 3)(70,Inf] \| 1.9193 \| 0.3392 \| 10.8605 \| 0.460980 \| \| t1sose_2000.fSelf-employed or employers \| 0.1542 \| 0.0213 \| 1.1183 \| 0.064403 \| \| t1sose_2000.fLower-level employees \| 1.1458 \| 0.7960 \| 1.6495 \| 0.463968 \| \| t1sose_2000.fManual workers \| 0.7873 \| 0.4976 \| 1.2456 \| 0.306966 \| \| t1sose_2000.fUnemployer \| 0.4513 \| 0.2111 \| 0.9646 \| 0.040076 \| \| t1sose_2000.fStudents \| 0.8722 \| 0.3724 \| 2.0429 \| 0.752895 \| \| t1sose_2000.fPensioners \| 0.4582 \| 0.1744 \| 1.2035 \| 0.113171 \| \| t1sose_2000.fUnknown \| 0.7066 \| 0.3323 \| 1.5022 \| 0.366743 \| \| statin.presc.blyes \| 0.3077 \| 0.0420 \| 2.2562 \| 0.246241 \| \| cN.teeth.124-27 \| 1.1819 \| 0.8006 \| 1.7448 \| 0.400476 \| \| cN.teeth.10-23 \| 1.3543 \| 0.6333 \| 2.8962 \| 0.434204 \| \| i.index.max1-2 \| 0.8053 \| 0.5464 \| 1.1871 \| 0.274143 \| \| i.index.max3-4 \| 0.7099 \| 0.4480 \| 1.1247 \| 0.144449 \| \| i.index.max>5 \| 0.8932 \| 0.5940 \| 1.3430 \| 0.587240 \| \| D.index.max1-2 \| 1.1057 \| 0.7556 \| 1.6181 \| 0.605062 \| \| D.index.max3-4 \| 1.3148 \| 0.8029 \| 2.1532 \| 0.276742 \| \| D.index.max>4 \| 1.3791 \| 0.7989 \| 2.3805 \| 0.248544 \| \| Relevel(DMF.index.max, ref = 3)0-13 \| 0.8643 \| 0.5734 \| 1.3027 \| 0.485968 \| \| Relevel(DMF.index.max, ref = 3)14-18 \| 0.7850 \| 0.5230 \| 1.1782 \| 0.242613 \| \| Relevel(DMF.index.max, ref = 3)>24 \| 0.6565 \| 0.4016 \| 1.0732 \| 0.093328 \| \| cpi1.max2 \| 0.8985 \| 0.5923 \| 1.3630 \| 0.614714 \| \| cpi1.max3-4 \| 0.3815 \| 0.2076 \| 0.7010 \| 0.001907 \| \| periodontitisYes \| 1.3618 \| 0.9718 \| 1.9082 \| 0.072810 \| \| cariesYes \| 0.9512 \| 0.6244 \| 1.4491 \| 0.815952 \| \| endoCariesYes \| 0.8899 \| 0.6055 \| 1.3077 \| 0.552433 \|   *Crohn disease*   \|  \| RR \| 2.5% \| 97.5% \| P \| \| --- \| --- \| --- \| --- \| --- \| \| sukupfemale \| 0.6803 \| 0.3476 \| 1.3313 \| 0.260745 \| \| Relevel(tage, list(young = 1:2, old = 3:5))old \| 0.6222 \| 0.1702 \| 2.2750 \| 0.473145 \| \| t1sose_2000.fSelf-employed or employers \| 0.0000 \| 0.0000 \| Inf \| 0.982763 \| \| t1sose_2000.fLower-level employees \| 1.3983 \| 0.6166 \| 3.1707 \| 0.422215 \| \| t1sose_2000.fManual workers \| 0.7998 \| 0.2769 \| 2.3107 \| 0.679899 \| \| t1sose_2000.fUnemployer \| 0.2996 \| 0.0371 \| 2.4180 \| 0.257940 \| \| t1sose_2000.fStudents \| 1.5692 \| 0.3368 \| 7.3123 \| 0.566074 \| \| t1sose_2000.fPensioners \| 0.4697 \| 0.0532 \| 4.1472 \| 0.496520 \| \| t1sose_2000.fUnknown \| 0.9576 \| 0.2039 \| 4.4962 \| 0.956210 \| \| cN.teeth.124-27 \| 1.5218 \| 0.6768 \| 3.4216 \| 0.309793 \| \| cN.teeth.10-23 \| 0.5869 \| 0.0647 \| 5.3215 \| 0.635678 \| \| i.index.max1-2 \| 0.4936 \| 0.1900 \| 1.2821 \| 0.147136 \| \| i.index.max3-4 \| 1.2636 \| 0.5386 \| 2.9646 \| 0.590701 \| \| i.index.max>5 \| 0.7441 \| 0.2965 \| 1.8678 \| 0.529101 \| \| D.index.max1-2 \| 0.7137 \| 0.2906 \| 1.7530 \| 0.461927 \| \| D.index.max3-4 \| 0.9872 \| 0.3014 \| 3.2337 \| 0.983000 \| \| D.index.max>4 \| 2.3778 \| 0.8374 \| 6.7519 \| 0.103807 \| \| Relevel(DMF.index.max, ref = 3)0-13 \| 0.9374 \| 0.3940 \| 2.2307 \| 0.883884 \| \| Relevel(DMF.index.max, ref = 3)14-18 \| 0.5748 \| 0.2196 \| 1.5047 \| 0.259418 \| \| Relevel(DMF.index.max, ref = 3)>24 \| 0.7007 \| 0.2469 \| 1.9886 \| 0.503922 \| \| cpi1.max2 \| 0.8202 \| 0.3318 \| 2.0276 \| 0.667730 \| \| cpi1.max3-4 \| 0.4839 \| 0.1368 \| 1.7116 \| 0.260080 \| \| periodontitisYes \| 1.1197 \| 0.5115 \| 2.4510 \| 0.777233 \| \| cariesYes \| 0.5690 \| 0.1892 \| 1.7114 \| 0.315594 \| \| endoCariesYes \| 0.7075 \| 0.3194 \| 1.5672 \| 0.393782 \|   *Ulcerative colitis*   \|  \| RR \| 2.5% \| 97.5% \| P \| \| --- \| --- \| --- \| --- \| --- \| \| sukupfemale \| 0.7469 \| 0.5329 \| 1.0468 \| 0.090216 \| \| Relevel(tage, ref = 3)(29,40] \| 2.4394 \| 1.0713 \| 5.5545 \| 0.033667 \| \| Relevel(tage, ref = 3)(40,50] \| 2.0976 \| 0.9256 \| 4.7538 \| 0.075953 \| \| Relevel(tage, ref = 3)(60,70] \| 6.0880 \| 1.9817 \| 18.7036 \| 0.001609 \| \| Relevel(tage, ref = 3)(70,Inf] \| 2.8572 \| 0.4599 \| 17.7521 \| 0.259973 \| \| t1sose_2000.fSelf-employed or employers \| 0.1883 \| 0.0258 \| 1.3726 \| 0.099472 \| \| t1sose_2000.fLower-level employees \| 1.0907 \| 0.7259 \| 1.6389 \| 0.675978 \| \| t1sose_2000.fManual workers \| 0.7842 \| 0.4715 \| 1.3043 \| 0.349095 \| \| t1sose_2000.fUnemployer \| 0.4858 \| 0.2146 \| 1.0998 \| 0.083306 \| \| t1sose_2000.fStudents \| 0.7152 \| 0.2549 \| 2.0063 \| 0.524187 \| \| t1sose_2000.fPensioners \| 0.4211 \| 0.1431 \| 1.2387 \| 0.116151 \| \| t1sose_2000.fUnknown \| 0.6513 \| 0.2740 \| 1.5480 \| 0.331680 \| \| statin.presc.blyes \| 0.3551 \| 0.0480 \| 2.6267 \| 0.310537 \| \| cN.teeth.124-27 \| 1.0986 \| 0.7038 \| 1.7148 \| 0.679077 \| \| cN.teeth.10-23 \| 1.5294 \| 0.6726 \| 3.4780 \| 0.310740 \| \| i.index.max1-2 \| 0.8922 \| 0.5815 \| 1.3690 \| 0.601636 \| \| i.index.max3-4 \| 0.5697 \| 0.3267 \| 0.9937 \| 0.047460 \| \| i.index.max>5 \| 0.9449 \| 0.5995 \| 1.4893 \| 0.807157 \| \| D.index.max1-2 \| 1.2151 \| 0.7953 \| 1.8564 \| 0.367701 \| \| D.index.max3-4 \| 1.4049 \| 0.8146 \| 2.4232 \| 0.221519 \| \| D.index.max>4 \| 1.1668 \| 0.6130 \| 2.2209 \| 0.638588 \| \| Relevel(DMF.index.max, ref = 3)0-13 \| 0.8678 \| 0.5466 \| 1.3778 \| 0.547740 \| \| Relevel(DMF.index.max, ref = 3)14-18 \| 0.8524 \| 0.5441 \| 1.3354 \| 0.485611 \| \| Relevel(DMF.index.max, ref = 3)>24 \| 0.6352 \| 0.3641 \| 1.1080 \| 0.109865 \| \| cpi1.max2 \| 0.9164 \| 0.5731 \| 1.4656 \| 0.715665 \| \| cpi1.max3-4 \| 0.3526 \| 0.1761 \| 0.7061 \| 0.003260 \| \| periodontitisYes \| 1.4214 \| 0.9780 \| 2.0658 \| 0.065309 \| \| cariesYes \| 1.0570 \| 0.6701 \| 1.6671 \| 0.811692 \| \| endoCariesYes \| 0.9564 \| 0.6152 \| 1.4868 \| 0.842925 \|   *Severe psychosis*   \|  \| RR \| 2.5% \| 97.5% \| P \| \| --- \| --- \| --- \| --- \| --- \| \| sukupfemale \| 1.1160 \| 0.9430 \| 1.3207 \| 0.201462 \| \| Relevel(tage, ref = 3)(29,40] \| 1.4671 \| 1.0807 \| 1.9917 \| 0.013992 \| \| Relevel(tage, ref = 3)(40,50] \| 1.4802 \| 1.1055 \| 1.9819 \| 0.008454 \| \| Relevel(tage, ref = 3)(60,70] \| 0.5748 \| 0.2995 \| 1.1033 \| 0.096016 \| \| Relevel(tage, ref = 3)(70,Inf] \| 0.7172 \| 0.4240 \| 1.2130 \| 0.215007 \| \| t1sose_2000.fSelf-employed or employers \| 1.9966 \| 1.2298 \| 3.2415 \| 0.005164 \| \| t1sose_2000.fLower-level employees \| 1.3127 \| 0.9955 \| 1.7311 \| 0.053895 \| \| t1sose_2000.fManual workers \| 1.4304 \| 1.0511 \| 1.9464 \| 0.022771 \| \| t1sose_2000.fUnemployer \| 3.5917 \| 2.6554 \| 4.8582 \| 0.000000 \| \| t1sose_2000.fStudents \| 3.0520 \| 2.0342 \| 4.5791 \| 0.000000 \| \| t1sose_2000.fPensioners \| 6.2941 \| 4.4116 \| 8.9799 \| 0.000000 \| \| t1sose_2000.fUnknown \| 2.5090 \| 1.7386 \| 3.6207 \| 0.000001 \| \| statin.presc.blyes \| 1.1413 \| 0.6948 \| 1.8747 \| 0.601779 \| \| cN.teeth.124-27 \| 1.1475 \| 0.9334 \| 1.4106 \| 0.191593 \| \| cN.teeth.10-23 \| 0.8550 \| 0.6048 \| 1.2086 \| 0.374997 \| \| i.index.max1-2 \| 1.1887 \| 0.9591 \| 1.4733 \| 0.114357 \| \| i.index.max3-4 \| 1.0901 \| 0.8491 \| 1.3995 \| 0.498550 \| \| i.index.max>5 \| 1.0082 \| 0.7910 \| 1.2851 \| 0.947436 \| \| D.index.max1-2 \| 0.9171 \| 0.7435 \| 1.1312 \| 0.418775 \| \| D.index.max3-4 \| 1.0754 \| 0.8224 \| 1.4062 \| 0.595221 \| \| D.index.max>4 \| 1.2711 \| 0.9659 \| 1.6728 \| 0.086836 \| \| Relevel(DMF.index.max, ref = 3)0-13 \| 1.1677 \| 0.9190 \| 1.4838 \| 0.204455 \| \| Relevel(DMF.index.max, ref = 3)14-18 \| 0.9762 \| 0.7705 \| 1.2369 \| 0.842116 \| \| Relevel(DMF.index.max, ref = 3)>24 \| 0.9903 \| 0.7758 \| 1.2642 \| 0.937890 \| \| cpi1.max2 \| 0.8842 \| 0.6896 \| 1.1335 \| 0.331443 \| \| cpi1.max3-4 \| 1.0498 \| 0.7891 \| 1.3965 \| 0.738764 \| \| periodontitisYes \| 1.0370 \| 0.8614 \| 1.2484 \| 0.701401 \| \| cariesYes \| 1.1174 \| 0.9077 \| 1.3756 \| 0.295321 \| \| endoCariesYes \| 1.1856 \| 0.9508 \| 1.4785 \| 0.130584 \| |
| --- | --- | --- | --- | --- | --- | --- | --- | --- | --- | --- | --- | --- | --- | --- | --- | --- | --- | --- | --- | --- | --- | --- | --- | --- | --- | --- | --- | --- | --- | --- | --- | --- | --- | --- | --- | --- | --- | --- | --- | --- | --- | --- | --- | --- | --- | --- | --- | --- | --- | --- | --- | --- | --- | --- | --- | --- | --- | --- | --- | --- | --- | --- | --- | --- | --- | --- | --- | --- | --- | --- | --- | --- | --- | --- | --- | --- | --- | --- | --- | --- | --- | --- | --- | --- | --- | --- | --- | --- | --- | --- | --- | --- | --- | --- | --- | --- | --- | --- | --- | --- | --- | --- | --- | --- | --- | --- | --- | --- | --- | --- | --- | --- | --- | --- | --- | --- | --- | --- | --- | --- | --- | --- | --- | --- | --- | --- | --- | --- | --- | --- | --- | --- | --- | --- | --- | --- | --- | --- | --- | --- | --- | --- | --- | --- | --- | --- | --- | --- | --- | --- | --- | --- | --- | --- | --- | --- | --- | --- | --- | --- | --- | --- | --- | --- | --- | --- | --- | --- | --- | --- | --- | --- | --- | --- | --- | --- | --- | --- | --- | --- | --- | --- | --- | --- | --- | --- | --- | --- | --- | --- | --- | --- | --- | --- | --- | --- | --- | --- | --- | --- | --- | --- | --- | --- | --- | --- | --- | --- | --- | --- | --- | --- | --- | --- | --- | --- | --- | --- | --- | --- | --- | --- | --- | --- | --- | --- | --- | --- | --- | --- | --- | --- | --- | --- | --- | --- | --- | --- | --- | --- | --- | --- | --- | --- | --- | --- | --- | --- | --- | --- | --- | --- | --- | --- | --- | --- | --- | --- | --- | --- | --- | --- | --- | --- | --- | --- | --- | --- | --- | --- | --- | --- | --- | --- | --- | --- | --- | --- | --- | --- | --- | --- | --- | --- | --- | --- | --- | --- | --- | --- | --- | --- | --- | --- | --- | --- | --- | --- | --- | --- | --- | --- | --- | --- | --- | --- | --- | --- | --- | --- | --- | --- | --- | --- | --- | --- | --- | --- | --- | --- | --- | --- | --- | --- | --- | --- | --- | --- | --- | --- | --- | --- | --- | --- | --- | --- | --- | --- | --- | --- | --- | --- | --- | --- | --- | --- | --- | --- | --- | --- | --- | --- | --- | --- | --- | --- | --- | --- | --- | --- | --- | --- | --- | --- | --- | --- | --- | --- | --- | --- | --- | --- | --- | --- | --- | --- | --- | --- | --- | --- | --- | --- | --- | --- | --- | --- | --- | --- | --- | --- | --- | --- | --- | --- | --- | --- | --- | --- | --- | --- | --- | --- | --- | --- | --- | --- | --- | --- | --- | --- | --- | --- | --- | --- | --- | --- | --- | --- | --- | --- | --- | --- | --- | --- | --- | --- | --- | --- | --- | --- | --- | --- | --- | --- | --- | --- | --- | --- | --- | --- | --- | --- | --- | --- | --- | --- | --- | --- | --- | --- | --- | --- | --- | --- | --- | --- | --- | --- | --- | --- | --- | --- | --- | --- | --- | --- | --- | --- | --- | --- | --- | --- | --- | --- | --- | --- | --- | --- | --- | --- | --- | --- | --- | --- | --- | --- | --- | --- | --- | --- | --- | --- | --- | --- | --- | --- | --- | --- | --- | --- | --- | --- | --- | --- | --- | --- | --- | --- | --- | --- | --- | --- | --- | --- | --- | --- | --- | --- | --- | --- | --- | --- | --- | --- | --- | --- | --- | --- | --- | --- | --- | --- | --- | --- | --- | --- | --- | --- | --- | --- | --- | --- | --- | --- | --- | --- | --- | --- | --- | --- | --- | --- | --- | --- | --- | --- | --- | --- | --- | --- | --- | --- | --- | --- | --- | --- | --- | --- | --- | --- | --- | --- | --- | --- | --- | --- | --- | --- | --- | --- | --- | --- | --- | --- | --- | --- | --- | --- | --- | --- | --- | --- | --- | --- | --- | --- | --- | --- | --- | --- | --- | --- | --- | --- | --- | --- | --- | --- | --- | --- | --- | --- | --- | --- | --- | --- | --- | --- | --- | --- | --- | --- | --- | --- | --- | --- | --- | --- | --- | --- | --- | --- | --- | --- | --- | --- | --- | --- | --- | --- | --- | --- | --- | --- | --- | --- | --- | --- | --- | --- | --- | --- | --- | --- | --- | --- | --- | --- | --- | --- | --- | --- | --- | --- | --- | --- | --- | --- | --- | --- | --- | --- | --- | --- | --- | --- | --- | --- | --- | --- | --- | --- | --- | --- | --- | --- | --- | --- | --- | --- | --- | --- | --- | --- | --- | --- | --- | --- | --- | --- | --- | --- | --- | --- | --- | --- | --- | --- | --- | --- | --- | --- | --- | --- | --- | --- | --- | --- | --- | --- | --- | --- | --- | --- | --- | --- | --- | --- | --- | --- | --- | --- | --- | --- | --- | --- | --- | --- | --- | --- | --- | --- | --- | --- | --- | --- | --- | --- | --- | --- | --- | --- | --- | --- | --- | --- | --- | --- | --- | --- | --- | --- | --- | --- | --- | --- | --- | --- | --- | --- | --- | --- | --- | --- | --- | --- | --- | --- | --- | --- | --- | --- | --- | --- | --- | --- | --- | --- | --- | --- | --- | --- | --- | --- | --- | --- | --- | --- | --- | --- | --- | --- | --- | --- | --- | --- | --- | --- | --- | --- | --- | --- | --- | --- | --- | --- | --- | --- | --- | --- | --- | --- | --- | --- | --- | --- | --- | --- | --- | --- | --- | --- | --- | --- | --- | --- | --- | --- | --- | --- | --- | --- | --- | --- | --- | --- | --- | --- | --- | --- | --- | --- | --- | --- | --- | --- | --- | --- | --- | --- | --- | --- | --- | --- | --- | --- | --- | --- | --- | --- | --- | --- | --- | --- | --- | --- | --- | --- | --- | --- | --- | --- | --- | --- | --- | --- | --- | --- | --- | --- | --- | --- | --- | --- | --- | --- | --- | --- | --- | --- | --- | --- | --- | --- | --- | --- | --- | --- | --- | --- | --- | --- | --- | --- | --- | --- | --- | --- | --- | --- | --- | --- | --- | --- | --- | --- | --- | --- | --- | --- | --- | --- | --- | --- | --- | --- | --- | --- | --- | --- | --- | --- | --- | --- | --- | --- | --- | --- | --- | --- | --- | --- | --- | --- | --- | --- | --- | --- | --- | --- | --- | --- | --- | --- | --- | --- | --- | --- | --- | --- | --- | --- | --- | --- | --- | --- | --- | --- | --- | --- | --- | --- | --- | --- | --- | --- | --- | --- | --- | --- | --- | --- | --- | --- | --- | --- | --- | --- | --- | --- | --- | --- | --- | --- | --- | --- | --- | --- | --- | --- | --- | --- | --- | --- | --- | --- | --- | --- | --- | --- | --- | --- | --- | --- | --- | --- | --- | --- | --- | --- |
